# Quantitative MRI of lipid content for assessing fetal adipose tissue development and brown-to-white fat conversion

**DOI:** 10.1186/s13244-026-02288-3

**Published:** 2026-04-25

**Authors:** Shuzhen Ma, Yuchen Liu, Chunran Yang, Hongbo Pu, Yangmei Pu, Zaihang Yin, Min Kang

**Affiliations:** 1 Department of Radiology, Sichuan Provincial Woman’s and Children’s Hospital, Chengdu, Sichuan China; 2 The Affiliated Women’s and Children’s Hospital of Chengdu Medical College, Chendu, Sichuan China; 3 GE HealthCare MR Research, Beijing, China

**Keywords:** Fetus, White adipose tissue, Magnetic resonance imaging, Fat fraction, Development

## Abstract

**Objective:**

Brown and white adipose tissues in different fetal regions undergo distinct developmental processes. This study aimed to quantitatively evaluate the fetal adipose tissue development of singleton fetuses.

**Materials and methods:**

A total of 78 participants were recruited, of whom 42 participants were included in the statistical analyses. Multi-echo water-fat separation (IDEAL-IQ) was performed, and proton density fat fraction (PDFF) and apparent transverse relaxation rate (R2*) values were measured for multiple anatomical regions, including cheeks, occiput, underjaw, neck, back, thorax, abdomen, and thighs. Correlations with gestational age were analyzed, and analysis of covariance (ANCOVA) was conducted by grouping participants into early (28–31 GW), mid (32–34 GW), and late (35–38 GW) gestational stages.

**Results:**

PDFF values in all fetal regions showed significant positive correlations with gestational age (*p* < 0.05). R2* values demonstrated region-specific patterns: no significant correlation was found for the back, while the cheeks showed a negative correlation; other regions showed significant correlations (*p* < 0.05). ANCOVA revealed significant differences in PDFF and R2* values across multiple regions among the early, mid, and late gestational groups (*p* < 0.01), indicating distinct stage- and region-specific characteristics of fetal fat deposition and iron metabolism.

**Conclusion:**

This study demonstrated that fetal PDFF increases globally with GW, while regional R2* patterns may reflect aspects of metabolic maturation. A change in R2* around approximately 32 weeks in the fetal cheek region may be indicative of an early transition from brown- to white-like adipose tissue. These findings indicate that IDEAL-IQ could be a useful tool for exploring fetal fat development.

**Critical relevance statement:**

This study provides quantitative MRI relevant to fetal brown-to-white fat conversion, suggesting a potential role for imaging markers in characterizing metabolic maturation in vivo. These findings may contribute to the development of noninvasive approaches for assessing fetal metabolic status and developmental variability.

**Key Points:**

- PDFF increased with gestational week, indicating progressive fetal fat maturation.
- Regional R2* patterns varied with gestational age, with changes in the cheek region around ~32 weeks that may be consistent with a transition from brown- to white-like adipose tissue.
- Fat development followed a “cheek → trunk → limb” sequence, showing spatial heterogeneity.

**Graphical Abstract:**

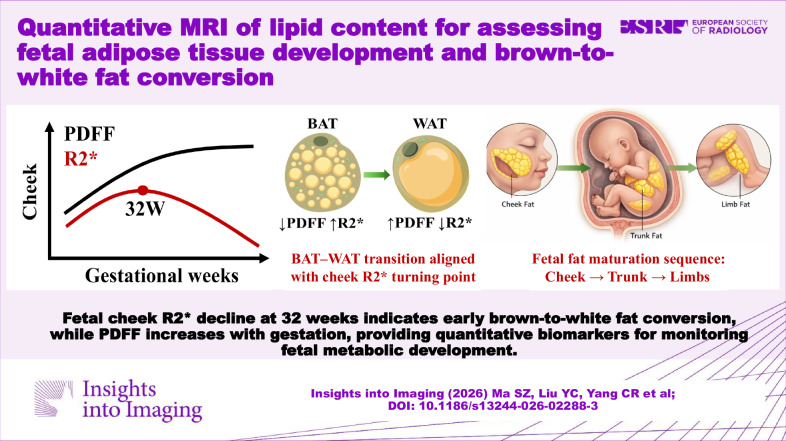

## Introduction

Fetal adipose tissue development reflects intrauterine nutritional status and is closely linked to postnatal energy balance and metabolic health. As an endocrine and metabolic organ, fetal adipose tissue contributes to energy storage, thermoregulation, and adipokine secretion (e.g., leptin, adiponectin), which support vascular, immune, and metabolic functions [1–3]. Brown adipose tissue (BAT), rich in mitochondria and highly oxidative, mediates non-shivering thermogenesis, whereas white adipose tissue (WAT) primarily stores energy [4]. During late gestation, BAT decreases and partially converts into WAT, potentially influencing long-term metabolic health [5]. Thus, fetal adipose tissue development may serve as an early biomarker for obesity and metabolic disorders [6–8]. However, systematic quantitative studies on the spatiotemporal pattern, regional heterogeneity, and timing of BAT-to-WAT conversion in human fetuses remain scarce.

Current assessments of fetal fat rely mainly on ultrasound and conventional MRI. Ultrasound can measure subcutaneous fat thickness but lacks the resolution to distinguish BAT (low lipid density, high perfusion) from WAT (high lipid density). Conventional T1/T2-weighted MRI provides morphological information on fat volume but lacks biophysical contrast for quantifying triglyceride fraction (WAT) or detecting mitochondrial and iron-related features (BAT). As a result, studies of BAT-to-WAT conversion remain largely indirect [9]. Moreover, systematic quantitative imaging studies on the ratio, maturation, and gestational evolution of BAT and WAT in human fetuses are still lacking.

The 3.0-T multi-echo chemical shift–encoded MRI technique (IDEAL-IQ) provides two quantitative biomarkers: (1) proton density fat fraction (PDFF), based on water–fat chemical shift separation, accurately quantifies the relative triglyceride content (WAT: PDFF > 80%; BAT: PDFF ~ 30–80%), with BAT generally exhibiting lower fat fraction than WAT [9–11]; (2) apparent transverse relaxation rate (R2*), derived from multi-echo gradient decay fitting, is sensitive to the concentration of paramagnetic substances (deoxyhemoglobin, cytochrome iron complexes) and positively correlates with the high mitochondrial density and vascularization of BAT (BAT: R2* > 100 s⁻¹; WAT: R2* < 50 s⁻¹) [9]. The higher field strength at 3.0 T enhances chemical shift sensitivity and temporal resolution, allowing regional analysis across fetal head/neck, trunk, and limbs, and supporting combined PDFF–R2* models to characterize BAT-to-WAT conversion [12].

In this study, we employed IDEAL-IQ to perform noninvasive, quantitative analysis of fetal adipose tissue during late gestation, aiming to identify and evaluate changes in BAT and WAT during fetal development and to determine the timing and regional characteristics of BAT-to-WAT conversion. We hypothesized that with advancing gestational week (GW), PDFF would progressively increase, whereas R2* might decrease, consistent with previously reported characteristics of BAT and WAT [5, 13]. Quantitative assessment of PDFF and R2* across different GWs may help to explore the temporal and spatial features of fetal adipose tissue development and provide imaging-based observations relevant to intrauterine nutritional status and metabolic maturation.

## Materials and methods

### Study population

This prospective study was approved by the Ethics Committee of Sichuan Provincial Maternity and Child Health Hospital (No. 20241031-419). Pregnant women who underwent MRI examinations at our institution between November 2024 and April 2025 were recruited. All participants provided written informed consent. Inclusion criteria were: no MRI contraindications, singleton pregnancy, and fetal thorax or abdominal anomalies detected by ultrasound. Pregnant women with metabolic disorders (e.g., diabetes, gestational diabetes), multiple pregnancies, or intrauterine growth restriction (IUGR) were excluded. A total of 78 non-diabetic singleton pregnancies between 28 and 38 GWs were enrolled. All fetuses were referred for MRI due to suspected thorax or abdominal anomalies on prenatal ultrasound; however, only 10 were confirmed to have structural abnormalities on MRI and were excluded. Of the remaining cases, 15 were excluded due to poor image quality (e.g., severe motion or excessive amniotic fluid), and 11 were excluded based on predefined design criteria, including maternal pre-pregnancy BMI outside the healthy range (18–24) and matching of maternal BMI, age, and gestational age. The final analysis included 42 fetuses, with gestational ages ranging from 28 to 38 weeks and maternal ages ranging from 23 to 41 years.

### MRI protocol and parameters

All MR examinations were performed on a 3.0-T MRI scanner (Premier: GE Healthcare) equipped with a 30-channel AIR coil and a spine coil. Subjects were positioned in a left lateral [14] decubitus or supine feet-first position and wore non-magnetic earphones for hearing protection. Scans were performed in Normal mode with strict control of the specific absorption rate (SAR) to below 2 W/kg [15, 16], and each scan was limited to within 30 min. Examinations could be terminated at any time if participants experienced discomfort. A multi-echo water-fat separation technique (IDEAL-IQ) was used for all scans, employing six asymmetric echoes and a multi-peak fat spectral model to correct for T2* decay and the complex distribution of fat peaks. Based on three-point asymmetric echo IDEAL technology, the six echo signals with different echo times were used to generate in-phase, out-of-phase, water, fat, fat fraction (PDFF), and R2* relaxation rate maps, providing accurate and reproducible quantification of fetal adipose tissue [17–19]. Imaging parameters included: field of view (FOV): 36 × 36 cm²; matrix: 140 × 140; slice thickness: 4 mm; number of excitations (NEXs): 0.5; flip angle: 3°; repetition time (TR)/echo time (TE): 6.2 ms/2.8 ms.

### ROI placement and quantification of PDFF and R2*

**Fig. 1:**
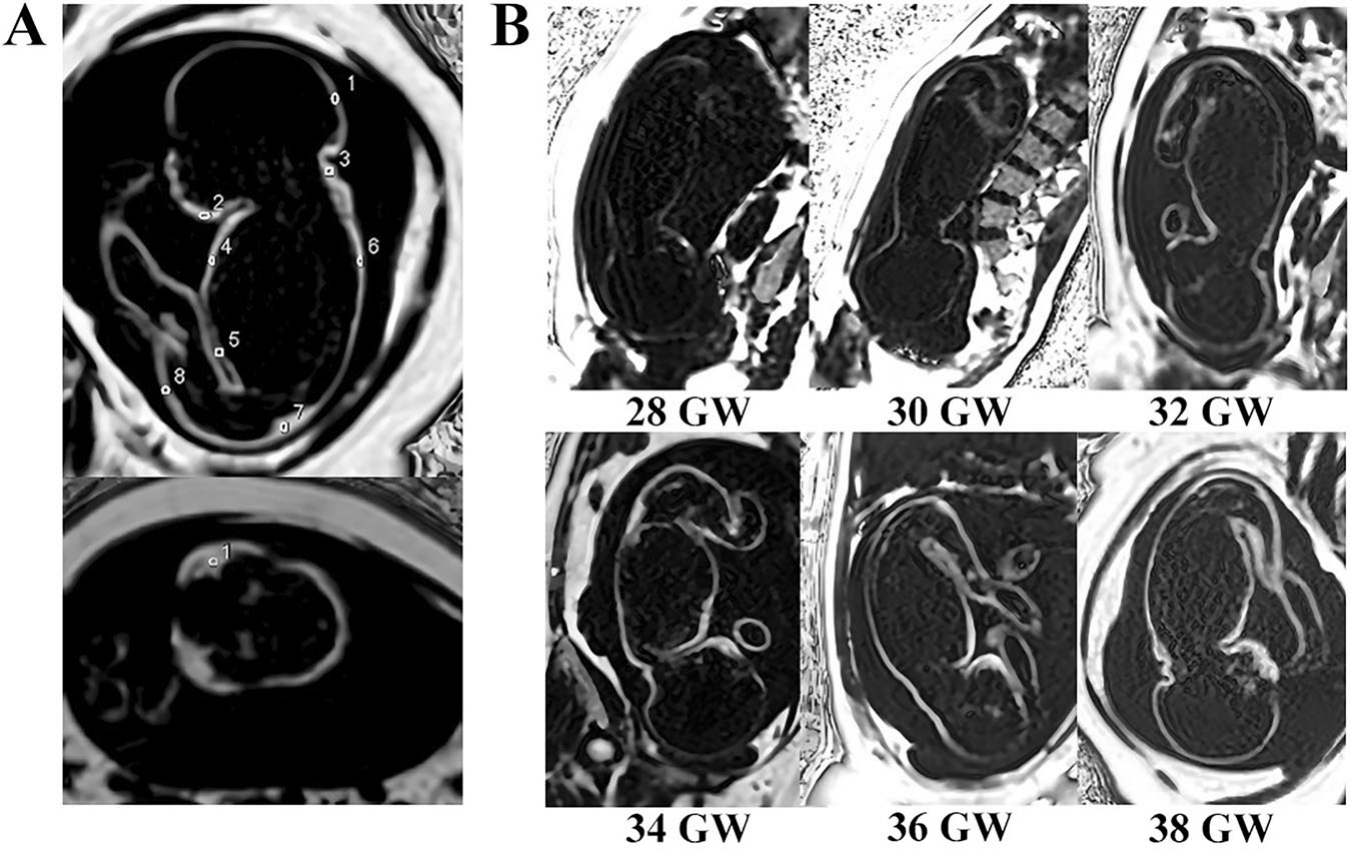
ROI localization and representative PDFF maps across gestational weeks. **A** ROI placement on PDFF maps: in the upper panel, labels 1–8 correspond to the occiput, chin, neck, back, thorax, abdomen, hip, and thigh; in the lower panel, label 1 corresponds to the cheeks. **B** Representative PDFF maps at 28, 30, 32, 34, 36, and 38 gestational weeks. GW, gestational week; PDFF, proton density fat fraction

Regions of interest (ROIs) were manually defined on anatomically consistent locations across subjects by two radiologists with more than five years of experience (M.S., Pu Y.) in both PDFF [20, 21] and R2* maps, including the cheeks, occiput, underjaw, posterior neck, back, thorax, abdomen, hip and thigh (Fig. 1). all ROIs were standardized to an area of approximately 10 mm² and was drawn on the mid-sagittal plane. Non-adipose structures, including bones, major vessels, and adjacent organs, were carefully avoided. Areas affected by visible susceptibility, fetal bone artifacts, or motion-related distortion were excluded from the ROIs. All ROIs were manually delineated using the GE AW workstation (GE Healthcare), first on the PDFF map and then applied to the corresponding locations on the R2* map. The mean value within each ROI was recorded as the PDFF and R2* measurement for that anatomical region. Segmentation was performed independently by both radiologists, cross-checked, and any discrepancies were resolved by consensus.

### Statistical analysis

Statistical analyses were performed using GraphPad Prism (version 10), SPSS (version 27) and MATLAB (2023b). Interobserver agreement for PDFF and R2* measurements between the two radiologists was evaluated using the intraclass correlation coefficient (ICC), calculated with a two-way mixed-effects model for absolute agreement. Continuous variables (GW, PDFF, R2*) were first tested for normality using the Shapiro–Wilk test. The homogeneity of regression slopes, a key assumption for analysis of covariance (ANCOVA), was evaluated by testing the interaction between the covariates and group for each feature (PDFF, R2*). ANCOVA was then applied to compare differences among the three GW groups (28–31 weeks, 32–34 weeks, 35–38 weeks), adjusting for potential confounders including maternal age, weight, height, BMI and abdominal circumference. For normally distributed data, Partial correlation analysis was then performed to assess the association between features (PDFF, R2*) and GW, controlling for the covariates, including maternal age, weight, height, BMI and abdominal circumference. Multiple comparisons for ANCOVA and partial correlation analysis were corrected using the Benjamini–Hochberg false discovery rate (FDR) procedure; *p*-values < 0.05 were considered statistically significant.

For the distinct heterogeneous patterns observed in the cheek region, where PDFF values were higher than in other regions, and R2* exhibited an initial increase followed by a decrease, non-linear regression models were applied to model the relationships between PDFF and GW (beta growth–then–decay model) as well as between R2* and GW (Lorentzian model). The models are defined as follows:

Beta growth–decay model:

$${{PDFF}}\left(t\right)={Y}_{m}\times \left(1+\frac{{T}_{e}-t}{{T}_{e}-{T}_{m}}\right)\times {\left(\frac{t}{{T}_{e}}\right)}^{\frac{{T}_{e}}{{T}_{e}-{T}_{m}}}$$

where *Y*_m_ is the peak PDFF value, *T*_m_ is the time of inflection point of the curve, and *T*_e_ is the time at which PDFF value peaks.

Lorentzian (Cauchy) model:

$${R2}^{* }\left(t\right)=\frac{{Y}_{m}}{1+{(\frac{t-{T}_{m}}{{T}_{e}})}^{2}}$$

where *Y*_m_ represents the R2* value, *T*_m_ is the time at peak R2*, and *T*_e_ is the time at half-width at half-maximum, reflecting the temporal extent of elevated R2*.

These models were selected to empirically describe the observed non-linear trend rather than to imply a specific biological mechanism. Inflection points and their 95% confidence intervals (CIs) were estimated using bootstrap resampling (*n* = 1000).

## Results

Demographic analysis showed no significant differences among the early-, mid-, and late-gestation groups (42 participants) in maternal age, height, weight, BMI or abdominal circumference (Table 1, *p* > 0.05). Measurement reproducibility was significant in PDFF (ICC > 0.9, *p* < 0.001) and R2* (ICC = 0.887, *p* < 0.001). Shapiro–Wilk tests indicated that GW, PDFF and R2* values in all regions were normally distributed (*p*-range: 0.120–0.886). All features yielded non-significant interaction *p*-values (range: 0.641–0.988), confirming the homogeneity of regression slopes required for ANCOVA.

**Table 1:** Demographic analysis

Groups	28–31 GW (*n* = 11)	32–34 GW (*n* = 18)	35–38 GW (*n* = 13)	*p*-value
Age (year)	29.45 ± 3.29	30.11 ± 4.53	30.30 ± 4.73	0.881
Height (cm)	159.54 ± 5.97	159.33 ± 4.10	157.61 ± 4.83	0.544
Weight (kg)	62.50 ± 8.50	60.99 ± 5.44	64.14 ± 9.36	0.531
BMI (kg/m^2^)	24.54 ± 2.98	24.01 ± 1.68	25.71 ± 2.64	0.156
Abdominal perimeter (cm)	93.63 ± 5.73	93.69 ± 4.84	97.30 ± 6.54	0.169

Data are presented as mean ± standard deviation. ANCOVA revealed no significant differences (*p* > 0.05) in age, height, weight, or abdominal perimeter of the pregnant women among the three gestational groups (28–31, 32–34, and 35–38 weeks), indicating comparable baseline characteristics

*GW* gestational week

ANCOVA revealed significant gestational group differences in PDFF for the cheeks, neck, thorax, back, hip, and thigh (Table 2, FDR-corrected *p* < 0.05), but not for the occiput, abdomen, and underjaw (Table 2, FDR-corrected *p* > 0.05). For R2*, no significant group differences were observed in the neck, back, thorax and hip (Table 2, *p* > 0.05), whereas other anatomical sites showed significant changes across gestational groups (Table 2, *p* < 0.05).

**Table 2:** Group differences of PDFF and R2*

	28–31 GW (*n* = 11)	32–34 GW (*n* = 18)	35–38 GW (*n* = 13)	BHFDR-corrected *p*
Cheeks PDFF (%)	63.73 ± 11.08	72.69 ± 10.73	78.85 ± 4.86	0.031
Cheeks R2* (s⁻¹)	44.71 ± 13.57	49.18 ± 14.84	31.02 ± 10.87	0.032
occiput PDFF (%)	13.74 ± 5.59	18.09 ± 6.38	20.47 ± 4.80	0.050
occiput R2* (s⁻¹)	25.71 ± 16.04	41.83 ± 15.10	51.25 ± 16.63	0.010
Neck PDFF (%)	23.28 ± 6.57	36.94 ± 10.49	37.78 ± 7.00	0.007
Neck R2* (s⁻¹)	32.80 ± 18.54	39.97 ± 18.52	49.53 ± 15.91	0.192
Underjaw PDFF (%)	31.63 ± 12.24	39.84 ± 11.31	41.42 ± 7.48	0.138
Underjaw R2* (s⁻¹)	35.52 ± 14.27	45.77 ± 16.94	60.25 ± 22.75	0.037
Thorax PDFF (%)	24.10 ± 8.78	31.92 ± 12.12	41.92 ± 8.38	0.021
Thorax R2* (s⁻¹)	38.30 ± 18.33	40.13 ± 14.26	52.72 ± 17.27	0.080
Back PDFF (%)	16.14 ± 9.69	21.62 ± 8.39	29.08 ± 7.65	0.037
Back R2* (s⁻¹)	32.75 ± 23.01	35.46 ± 16.30	42.65 ± 18.02	0.697
Abdomen PDFF (%)	17.45 ± 5.86	21.68 ± 6.16	29.22 ± 8.75	0.016
Abdomen R2* (s⁻¹)	26.24 ± 12.23	47.43 ± 24.10	41.60 ± 14.63	0.050
Hip PDFF (%)	35.53 ± 10.78	46.95 ± 13.43	52.98 ± 14.78	0.050
Hip R2* (s⁻¹)	41.86 ± 21.69	42.05 ± 19.79	47.69 ± 20.05	0.697
Thigh PDFF (%)	23.91 ± 7.12	35.15 ± 8.87	42.50 ± 11.18	0.007
Thigh R2* (s⁻¹)	27.16 ± 19.48	44.84 ± 16.12	49.16 ± 13.43	0.037

Quantitative measurements (mean ± SD) of proton density fat fraction (PDFF, %) and apparent transverse relaxation rate (R2, s⁻¹) across fetal anatomical regions for the three gestational age groups. *p*-values are derived from an analysis of covariance (ANCOVA) comparing the three groups

*GW* gestational week, *PDFF* proton density fat fraction, R2* apparent transverse relaxation rate

**Fig. 2:**
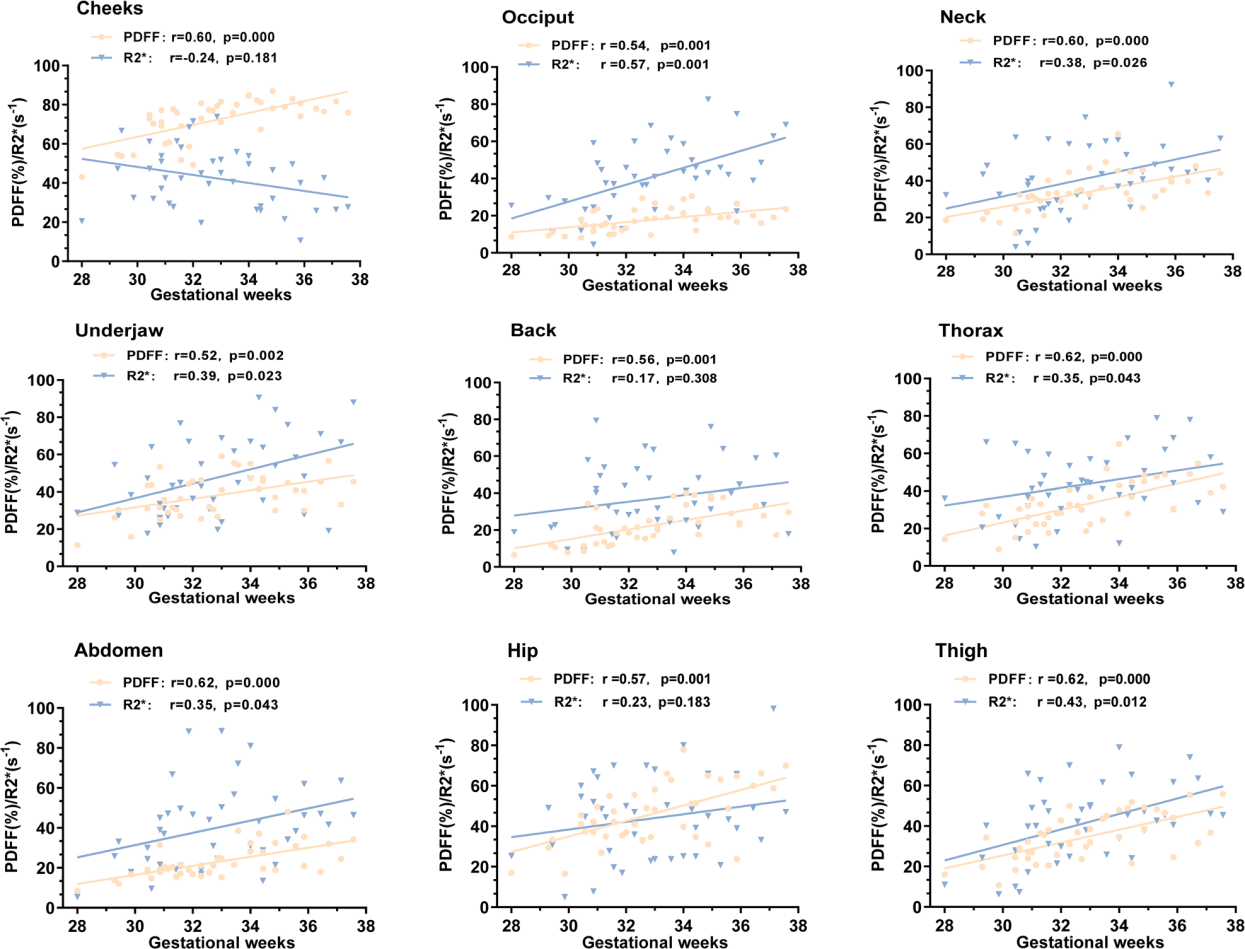
Scatter plots illustrating the associations between GW and both PDFF (%) and R2 (s⁻¹) values across different anatomical regions. The panels are arranged from left to right and top to bottom as follows: the first row shows cheeks, occiput, and neck; the second row shows underjaw, back, and thorax; and the third row shows abdomen, hip, and thigh. Significant positive correlations were observed between GW and PDFF in all regions (*p* < 0.05). GW, gestational week; PDFF, proton density fat fraction; R2*, apparent transverse relaxation rate

Partial correlation analysis demonstrated significant positive correlations between GW and PDFF in all regions (Fig. 2 and Table 3, FDR-corrected *p* < 0.05), indicating an overall increase in PDFF with advancing gestation. R2* correlations with GW varied by region: no significant associations were found in the cheeks, back, and hip (FDR-corrected *p* > 0.05); a weak negative correlation was observed in the cheeks (*r* = –0.24, FDR-corrected *p* = 0.181); and significant positive correlations were found in all other measured regions (FDR-corrected *p* < 0.05).

**Table 3:** Partial correlation analysis between GW and PDFF (%), R2* (s⁻¹)

Region	*R*	BHFDR-corrected *p*
Cheeks PDFF (%)	0.60	0.000
Cheeks R2* (s⁻¹)	−0.24	0.181
occiput PDFF (%)	0.54	0.001
occiput R2* (s⁻¹)	0.57	0.001
Neck PDFF (%)	0.60	0.000
Neck R2* (s⁻¹)	0.38	0.026
Underjaw PDFF (%)	0.52	0.002
Underjaw R2* (s⁻¹)	0.39	0.023
Thorax PDFF (%)	0.62	0.000
Thorax R2* (s⁻¹)	0.35	0.043
Back PDFF (%)	0.56	0.001
Back R2* (s⁻¹)	0.17	0.308
Abdomen PDFF (%)	0.62	0.000
Abdomen R2* (s⁻¹)	0.35	0.043
Hip PDFF (%)	0.57	0.001
Hip R2* (s⁻¹)	0.23	0.183
Thigh PDFF (%)	0.62	0.000
Thigh R2* (s⁻¹)	0.43	0.012

Pearson correlation coefficients (*r*) and false discovery rate (FDR)-corrected *p*-values for the associations between GW and PDFF/R2* in each fetal anatomical region

*GW* gestational week, *PDFF* proton density fat fraction, R2* apparent transverse relaxation rate

R2* is specific scientific term, and 35.39 (0–37.92)* means the confidence interval touches or exceeds the valid bounds of the parameter

**Fig. 3:**
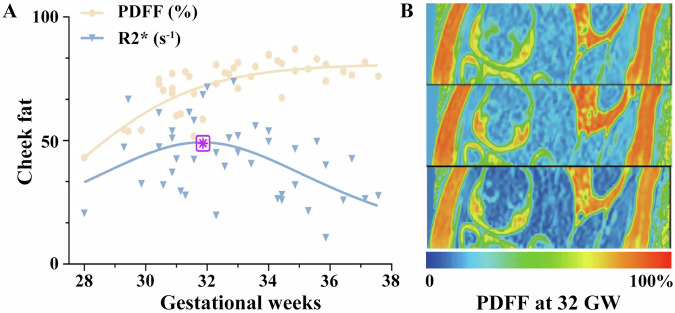
Nonlinear developmental patterns of fetal cheek adipose tissue. **A** Nonlinear fitting of cheek PDFF was performed using the least-squares method with a beta growth–decay model (*R*² = 0.53). The trend of cheek R2* values was modeled with a Lorentzian (Cauchy) function (*R*² = 0.22), showing a standardized peak (Tm) at 31.8 gestational weeks. **B** Representative color-coded PDF map of the fetal cheek fat pad at 32 gestational weeks, with PDF values ranging from 0–100%. GW, gestational week; PDFF, proton density fat fraction; R2*, apparent transverse relaxation rate

Figure 3 showed the nonlinear modeling of cheek fat development, indicating that PDFF plateaued in late gestation, whereas R2* exhibited an inflection point at approximately 32 weeks (31.8 weeks, 95% CI: 30.9–32.6 weeks), followed by a decline. This pattern suggests that BAT-to-WAT conversion in the cheek region begins around 32 weeks of gestation, making it the earliest maturing fetal fat depot. Table 4 showed the coefficients of the fitted models for PDFF and R2*.

**Table 4:** Model fitting coefficients for PDFF and R2* in the fetal cheek fat pad

Coefficients	Cheek PDFF	Cheek R2*
Model	beta growth then decay	Lorentzian (Cauchy)
Ym	80.99 (77.26–84.83)	49.27 (43.02–56.10)
Tm	28.72 (26.56–29.71)	31.83 (28.86–32.84)
Te	35.39 (0–37.92)*	5.485 (3.580–12.06)
R²	0.5388	0.2238
Root mean square error (Sy.x)	7.565	13.81
AICc	175.9	226.5

Coefficients and goodness-of-fit metrics for the nonlinear models applied to characterize the development of fetal cheek adipose tissue. PDFF was fitted with a beta growth-then-decay model, and R2* was fitted with a Lorentzian (Cauchy) model

*PDFF* proton density fat fraction, R2* apparent transverse relaxation rate

R2* is specific scientific term, and 35.39 (0–37.92)* means the confidence interval touches or exceeds the valid bounds of the parameter

## Discussion

In this study, we employed the 3.0 T MRI IDEAL-IQ sequence to perform a systematic quantitative assessment of lipid accumulation (PDFF) and metabolic activity (R2*) across nine anatomical regions in singleton fetuses during late gestation. Our findings provide novel insights into the spatiotemporal dynamics of fetal adipose tissue development and the transition from brown adipose tissue (BAT) to white adipose tissue (WAT). We observed that PDFF increased significantly with advancing GW in most regions, whereas R2* demonstrated region-specific patterns, with some regions showing an initial rise followed by a decline. These R2* patterns may reflect, in part, metabolic changes associated with the maturation of adipose tissue, with the cheek region showing an inflection point between 31 and 33 weeks that is suggestive of an early transition from BAT-like to WAT-like tissue, highlighting a potential window of fetal fat maturation.

### Regional heterogeneity results from ANCOVA

ANCOVA revealed marked regional heterogeneity in fetal adipose tissue development across gestational stages. Cheek fat: maturation occurred as early as mid-gestation (approximately 14.5–17 weeks), with PDFF values approaching those of neonates, and an R2* inflection point emerging around 32 weeks, suggesting that BAT-to-WAT conversion in this region is completed earliest. Abdominal and gluteal fat: PDFF increased rapidly during late gestation, consistent with previous reports that “late pregnancy is dominated by fat accumulation” [22, 23]. However, no distinct R2* inflection point was observed, possibly indicating delayed or postnatal BAT conversion. Limb fat: development lagged behind, with slower PDFF increases, which may reflect its secondary role in energy storage.

Giza et al proposed a “cheek → limbs → trunk” developmental sequence, inferring that facial subcutaneous fat appears first and subsequently extends distally to the extremities [13]. In contrast, the present study, based on quantitative analysis of both PDFF and R2* across multiple regions, demonstrated a “cheek → trunk → limbs” progression, wherein facial adipose tissue accumulation and metabolic activity emerge first, followed by the trunk, while the limbs show delayed deposition. This discrepancy may arise from methodological differences: Giza’s study averaged adipose tissue across entire anatomical regions, which could be confounded by motion artifacts, whereas the present study selectively analyzed localized ROIs free of motion interference.

Our findings are consistent with prior histological and imaging studies [5, 24], as well as with the known migration patterns of embryonic mesenchymal cells [5]. Collectively, these results highlight regional differences in fetal blood supply, thermogenic demand, and energy allocation. The preferential growth of facial and truncal fat supports central thermoregulation and vital organ protection, whereas the delayed development of distal limb fat reflects a staged and hierarchical distribution of fetal energy resources.

### Partial correlation analysis between GW and PDFF, R2*

The correlations between GW and PDFF, R2*, demonstrated distinct spatial patterns. PDFF was significantly positively correlated with GW in all regions, with the strongest associations observed in the thorax, cheeks, and thigh. This finding indicates that lipid accumulation in late gestation occurs predominantly in the face, trunk, and proximal limbs. This distribution pattern aligns with previous ultrasound and autopsy observations and supports the “central-to-peripheral” gradient of lipid deposition, whereby adipose tissue deposition begins in the face and subsequently extends to the trunk and large extremities [22].

In contrast, R2* exhibited marked regional heterogeneity in its correlation with GW. Significant positive correlations were observed in the posterior neck, chin, abdomen, and thighs, suggesting that these regions maintain increased concentrations of paramagnetic substances (e.g., deoxyhemoglobin, iron) during late gestation, reflecting sustained metabolic activity and adequate perfusion. Conversely, cheek R2* values demonstrated a weak negative correlation with GW, implying that this BAT-enriched region may undergo brown-to-white fat conversion in late gestation, accompanied by reduced mitochondrial density and vascularization, leading to diminished paramagnetic content and declining R2* values [23].

For other regions, including the occiput, back, hip and thorax, R2* showed no significant or only weak correlations with GW. This may be attributable to the relatively small volume of subcutaneous fat in the forehead and limited BAT distribution, while in the back and thorax—both potential BAT depots—high vascularization and layered structures, coupled with motion artifacts and stable developmental patterns, may reduce the sensitivity of R2* to subtle metabolic changes compared with regions such as the cheeks or posterior neck [25].

### Nonlinear developmental patterns of fetal cheek fat

Nonlinear analysis of cheek adipose tissue development demonstrated an R2* inflection point at approximately 32 weeks of gestation (32.8 ± 1.5 weeks), followed by a declining trend (Fig. 3). This pattern is consistent with the expected metabolic characteristics of BAT [26]. The observed decline in R2* was accompanied by a slower increase in PDFF during late gestation. Takashi Abe reported that the fat fraction of the buccal fat pad increases from 72.6% at birth to approximately 90% at one month of age [27]. In the present study, the mean mid-to-late gestational PDFF (73.13 ± 8.78) was nearly identical to neonatal values reported in the literature (72.2%), indicating that cheek adipose tissue had already reached a relatively mature stage by this period [4, 27].

**Fig. 4:**
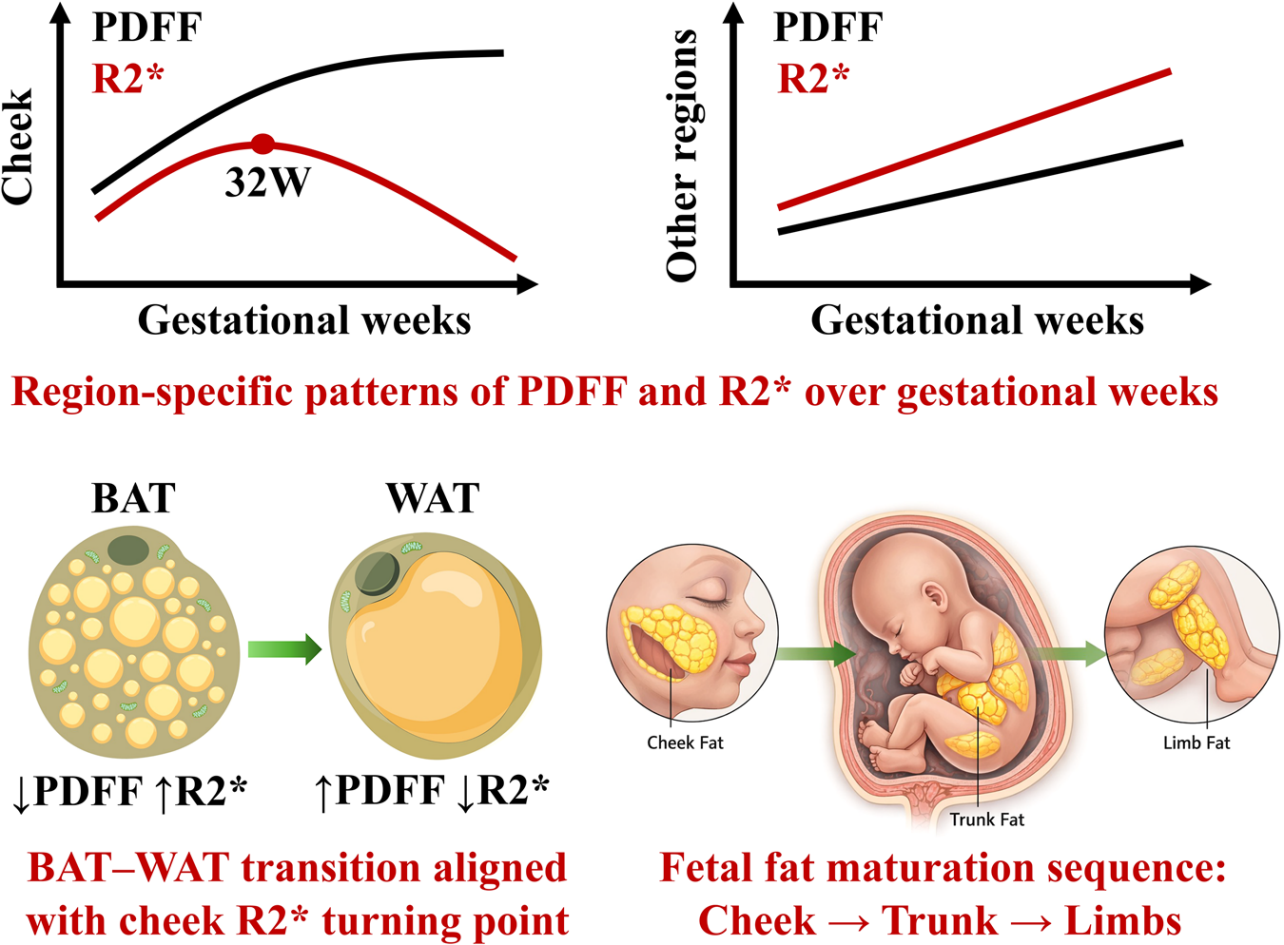
Diagram illustrating region-specific patterns of fetal fat development. Upper panels show schematic trajectories of PDFF (black) and R2* (red) across gestational weeks, with R2* increasing and then declining in the cheek region, in contrast to the predominantly increasing trends observed in other regions. Lower left panels depict the biological transition from BAT to WAT, characterized by increasing PDFF and decreasing R2*. Lower right panels illustrate the proposed spatial maturation sequence of fetal adipose tissue, progressing from the cheek to the trunk and subsequently to the limbs. Together, the schematic summarizes how regional fat maturation and the BAT–WAT transition may be reflected by PDFF and R2* changes during late gestation. GW, gestational week; PDFF, proton density fat fraction; R2*, apparent transverse relaxation rate; BAT, brown adipose tissue; WAT, white adipose tissue

Biologically, BAT exhibits higher R2* values than WAT due to its dense mitochondrial content, expression of uncoupling protein 1 (UCP1), elevated iron levels, and rich capillary network [28, 29]. The observed decline in R2* during the BAT-to-WAT transition is consistent with reported reductions in mitochondrial density of 40–60% [28]. As adipose tissue converts to WAT, mitochondrial density decreases, lipid droplets fuse, and vascular remodeling occurs, collectively leading to reduced R2* values. This transition is accompanied by rising levels of adipokines such as adiponectin and leptin, which promote lipid storage and metabolic regulation [30]. Notably, adiponectin is produced by both adipocytes and vascular cells during fetal life, but exclusively by adipocytes after birth [31]. These findings are consistent with previous imaging and histological reports. For example, Eléonore et al described T1-weighted signal changes between 26 and 33 weeks of gestation [32], and Hu et al suggested that BAT-to-WAT conversion continues beyond birth into childhood [22, 33, 34]. We note, however, that R2* is influenced by multiple factors, including iron content, perfusion, susceptibility inhomogeneities, and deoxyhemoglobin levels. In the absence of complementary modalities such as magnetic resonance spectroscopy (MRS) or perfusion imaging, the observed decline in cheek R2* could be interpreted as suggestive of BAT-to-WAT conversion rather than definitive evidence (Fig. 4).

### Limitations

Several limitations should be acknowledged. First, the sample size was relatively small, particularly in the late-gestation group, which may have limited our ability to detect inflection points in some regions. Second, although strict image-quality control and predefined eligibility criteria were applied, the relatively high exclusion rate may introduce potential selection bias and limit the generalizability of our findings. Third, detailed maternal glycemic indices were not available for all participants, although all enrolled subjects were metabolically healthy with normal blood glucose levels and no history of diabetes. Finally, the absence of MRS precluded direct assessment of fatty acid composition changes; incorporating MRS and multi-parametric MRI could further elucidate the biochemical mechanisms underlying BAT-to-WAT conversion. Future work integrating automated segmentation and multi-modal MRI could further refine these assessments and expand their clinical utility.

## Conclusion

This study systematically characterizes the spatiotemporal patterns of PDFF and R2* across multiple fetal adipose tissue depots during late gestation. Our findings indicate that PDFF and R2* provide indirect and complementary quantitative information for assessing fetal adipose tissue maturation, with the cheek region’s R2* inflection point suggestive of an early transition from BAT-like to WAT-like tissue. While R2* is influenced by multiple factors and is not a BAT-specific biomarker, its combination with PDFF offers a valuable approach for evaluating regional fat development. This imaging strategy provides an objective framework for investigating fetal fat maturation and may inform future studies on the early identification and management of metabolic health risks during fetal development.

## Data Availability

The datasets used and/or analyzed during the current study are available from the corresponding author on reasonable request.

## Abbreviations

ANCOVA: Analysis of covariance
BAT: Brown adipose tissue
BMI: Body mass index
CI: Confidence interval
FDR: False discovery rate
GW: Gestational week
ICC: Intraclass correlation coefficient
IDEAL-IQ: Iterative decomposition of water and fat with echo asymmetry and least-squares estimation—quantitative
MRI: Magnetic resonance imaging
MRS: Magnetic resonance spectroscopy
PDFF: Proton density fat fraction
R2*: Apparent transverse relaxation rate
ROI: Region of interest
WAT: White adipose tissue
